# Syndrome of inappropiate antidiuretic hormone secretion (SIADH) secondary to sertraline: case report and literature review

**DOI:** 10.1192/j.eurpsy.2023.1173

**Published:** 2023-07-19

**Authors:** C. Cardenes-Moreno, S. Yelmo-Cruz, I. Perez-Sagaseta, J. J. Tascon-Cervera, J. Dorta-Gonzalez, A. Crisostomo-Siverio, L. Torres-Tejera, M. Paniagua-Gonzalez, S. Canessa, M. R. Cejas-Mendez

**Affiliations:** Psychiatry, Hospital Universitario de Canarias, La Laguna, Spain

## Abstract

**Introduction:**

Currently, in addition to their frequent use in community medicine, the use of antidepressants is a fundamental pillar of pharmacological treatments used in psychiatry. Due to this frequent use, we must be aware of the possible side effects, in particular the SIADH produced in this clinical case by SSRIs. There are already described cases of this association including other antidepressants and many different types of drugs.

**Objectives:**

To review the current literature on the management of this pathology when it is secondary to the use of frequently used drugs such as SSRIs.

**Methods:**

We report the case of a 64-year-old woman hospitalised in the psychiatric department for malnutrition secondary to unspecified eating disorder (ED). During admission, treatment with sertraline was started with ascending doses up to 100mg, subsequently producing slight edema with the following analytical results: plasma Na: 123 mEq/L (135-145), plasma osmolarity: 250 mOsm/kg (275-300), urinary Na: 174 mEq/L (>40), fulfilling diagnostic criteria for SIADH.

Afterwards, we reduced sertraline until discontinuation and started treatment with water restriction and urea (30 grams/24 hours) during admission and after discharge. During admission, we observed disappearance of the edema and partial improvement of the analytical values (Na:131 mEq/L), which were normalised with home treatment of daily urea.

**Results:**

The precise prevalence of SIADH from the use of SSRIs is unknown, it is known that patients older than 65 are at higher risk of developing severe hyponatraemia in the first 5 weeks after initiation. Similarly, treatment with water and urea restriction, together with discontinuation of SSRIs, appears to be sufficient.

**Conclusions:**

SSRIs can cause SIADH a reversible but potentially life-threatening pathology, and we need to be aware of this possibility especially in the older population and being able to handle it

**Disclosure of Interest:**

None Declared

